# Azure Kinect for joint range of motion analysis: A validity and reliability evaluation in elite female weightlifters

**DOI:** 10.1371/journal.pone.0334890

**Published:** 2025-11-21

**Authors:** Serkan Örücü, Kenan Erdağı, Bülent Işık, Erkan Özbay, Usame Ömer Osmanoğlu

**Affiliations:** 1 Karamanoğlu Mehmetbey University, Vocational School of Technical Sciences, Computer Technologies Program, Karaman, Turkey; 2 Department of Physical Education and Sports, Ahmet Keleşoğlu Faculty of Education, Necmettin Erbakan University, Konya, Turkey; 3 Department of Physiology, Medical School, University of Karamanoğlu Mehmetbey, Karaman, Turkey; 4 Karamanoğlu Mehmetbey University Vocational School of Health, Karaman, Turkey; 5 Departments of Biostatistics, Medical School, University of Karamanoğlu Mehmetbey, Karaman, Turkey; Indian Institute of Technology Patna, INDIA

## Abstract

Kinect is a markerless, portable, and affordable motion analysis tool used in clinical, rehabilitation, and sports settings. This study aimed to assess upper limb (shoulder and elbow) and cervical joint angles in elite female weightlifters using Azure Kinect and a digital goniometer, and to evaluate the Kinect’s validity and reliability. Joint angles were measured in elite female weightlifters (n = 21) using both a digital goniometer and Azure Kinect, with three repetitions per movement under standardized conditions (within a stabilization cage). Mean values were used for analysis. Statistical analysis included descriptive metrics and non-parametric tests. Tool comparisons were conducted using the Wilcoxon signed-rank test, Spearman’s correlation coefficient, and Bland–Altman plots. Reliability was evaluated through the Intraclass Correlation Coefficient (ICC), Coefficient of Variation (CV), and Coefficient of Repeatability (CR), with statistical significance set at p < 0.05. Most joint angle measurements showed no statistically significant differences between the digital goniometer and Azure Kinect (p > 0.05), although small discrepancies appeared in some movements. High correlation coefficients (r = 0.82–0.99) and strong agreement based on Intraclass Correlation Coefficient (ICC = 0.97–0.99) were observed. Bland–Altman analysis revealed minimal systematic bias and narrow confidence intervals. Comparable values in Coefficient of Variation (CV) and Coefficient of Repeatability (CR) indicated high stability and repeatability for both tools. Overall, results support Azure Kinect as a valid and reliable alternative to traditional digital goniometry for assessing cervical and upper extremity joint range of motion.

## Introduction

Measuring joint range of motion (ROM) during both static and dynamic, as well as passive and active, movements are considered a key aspect of musculoskeletal assessment in clinical practice. These measurements help assess joint function, reveal potential asymmetries, and support objective tracking of treatment progress. In sports science, certain strength and conditioning coaches also make use of ROM evaluations as part of their performance monitoring strategies [1].

The most commonly used ROM measurement tool in clinical settings is the universal goniometer (UG) [2]. The UG consists of a circular protractor with a 360-degree scale and two transparent arms. During measurement, the center of the device is aligned with the joint axis, the fixed arm is placed along the static limb, and the movable arm follows the dynamic limb. It has been stated that the UG is regarded as the “gold standard” in the literature due to its affordability, portability, and ease of use [3,4]. It has also been reported to demonstrate high reliability, particularly in large joints such as the shoulder and knee [5–7]. However, its measurement accuracy can be influenced by several factors, including the practitioner’s level of experience, bimanual coordination, and proper joint stabilization [8,9]. Additionally, since the UG allows measurement only within a single plane and static positions, it remains inadequate for assessing dynamic movement [10].

In recent years, a range of technological solutions including electrogoniometers, digital imaging systems, 3D motion analysis systems, and particularly depth sensor devices—have gained increasing attention as a means to address these limitations [7,9–14].

Microsoft Kinect is a low-cost, markerless 3D depth sensor capable of tracking body segments in real time using infrared (IR) projection combined with an IR camera. This device has been integrated with software across diverse fields including medicine, biomechanics, sports, and industry for the monitoring, evaluation, and analysis of human movement.

Several studies have demonstrated that data obtained using Kinect exhibits high reliability, particularly in shoulder flexion and abduction movements. Notably, in a comparative analysis with the HumanTrak system, the Kinect-based measurement approach showed strong intra-rater and inter-rater reliability, with minimal measurement error [15].

Likewise, a separate study involving shoulder flexion, abduction, and external rotation reported that Kinect-based measurements showed a high correlation with traditional goniometric methods. The intraclass correlation coefficients (ICC) ranged between 0.864 and 0.942, while Cohen’s kappa values were between 0.88 and 1.0 [16]. Based on these results, the Kinect system has been considered a viable alternative for home-based rehabilitation and virtual exercise therapy. When compared with the Vicon motion analysis system, it demonstrated an average error rate of less than 10% in upper extremity joint angle measurements. These findings suggest that Kinect, due to its affordability and accessibility, holds promise for objective, quantitative assessments in home environments [17]. Furthermore, studies evaluating its validity and reliability in rehabilitation, posture, and balance analysis underscore its potential in clinical applications [18,19].

The accuracy of joint center estimation has been enhanced by calculating the lengths and movement directions of body segments using RGB-D (Red-Green-Blue plus Depth) data. This advancement has expanded the applicability of the system beyond ROM measurements, enabling its use in posture, gait, and balance assessments. Although Kinect’s non-anthropometric structure may cause a deviation of 2–8 cm in estimating arm and forearm lengths, adjustments made through integrated RGB and depth data reduced this variance by 72% and led to a 2° improvement in shoulder flexion accuracy [20].

Using three-dimensional joint coordinates obtained via the Kinect sensor, vector angles between joints can be computed, allowing for numerical estimation of joint control angles in body mechanics analysis [21]. Microsoft Kinect systems have demonstrated strong validity and reliability in studies focused on shoulder and elbow motion. In a study by Çubukçu et al. [22], ICC values ranging from 0.85 to 0.96 were reported for shoulder flexion, abduction, internal rotation, and external rotation measured using Kinect V2. Additionally, the average difference in abduction measurements between Kinect and a clinical goniometer was 2.83°, with limits of agreement (LoA) ranging from −6.25° to +11.91°, suggesting that the system offers acceptable measurement accuracy [22].

Similarly, a study by Bertram et al. [23] found that elbow and wrist measurements performed using Azure Kinect correlated highly with those obtained from a marker-based system (Qualisys). ICC values ranged from 0.84 to 0.99, while the mean root mean square error (RMSE) was between 20 and 33 mm. These results indicate that Azure Kinect serves as a reliable and reproducible tool for upper extremity motion analysis [23].

Haas et al. [24] demonstrated that a Kinect-based system enables physiotherapists to design patient-specific exercise programs and monitor exercise form directly through the software. This approach not only increases motivation for both clinicians and patients but also helps reduce injury risk by promoting proper posture. Similarly, Chen and Wei [21] developed real-time motion tracking, scoring, and body position analysis systems for sports science education using image processing algorithms based on Microsoft Kinect. The potential of Kinect as a cost-effective motion analysis tool has been emphasized in both sports training and large-scale physical activity applications [21]. Additionally, sensor-based systems have been applied in areas such as performance analysis, technical error correction, and posture optimization, thereby contributing to improved training quality and competitive performance among athletes [25].

Despite its growing use, the application of Kinect technology in elite athletes, particularly in sports requiring precise upper extremity control, such as weightlifting, remains limited. This limitation stems from both the lack of comprehensive validity and reliability studies within athletic populations and the insufficient integration of the technology into performance-driven systems. Nonetheless, some weightlifting specific studies have utilized the Kinect sensor to analyze biomechanical parameters such as bar velocity, knee angles, and movement patterns. Real-time data processing has been shown to support the identification of technical errors and contribute to enhanced training quality [26].

Research in this area highlights the broad potential of Kinect technology across both medical and sports domains. Nevertheless, further studies are required to establish the validity and reliability of Kinect-based measurement systems specifically in elite athletes, particularly in disciplines like weightlifting where upper extremity mobility plays a critical role. Moreover, the literature offers limited insight into joint range of motion analyses among female weightlifters, a population that is both underrepresented and characterized by high training experience. Evaluating this specific group could yield valuable contributions to scientific understanding and practical applications in athletic training.

Accordingly, this study aimed to evaluate the validity and reliability of an Azure Kinect-based system by comparing joint angle measurements obtained from elite female weightlifters using both Azure Kinect and a digital goniometer. The assessment focused on upper extremity (shoulder and elbow) and cervical region movements, including cervical flexion/extension, shoulder flexion/extension, abduction, internal/external rotation, and elbow flexion.

In this context, the main hypothesis of the study is that joint angle measurements specifically cervical flexion/extension, shoulder flexion/extension, abduction, internal/external rotation, and elbow flexion obtained using Azure Kinect will strongly correlate with those from a digital goniometer. Accordingly, the Kinect system is expected to serve as a valid and reliable tool for assessing upper extremity and cervical joint mobility in elite athletes.

## Materials and methods

### Participants and data collection

This study involved 21 elite female athletes from the Turkish Women’s National Weightlifting Team, all of whom were healthy and in preparation for upcoming international competitions. A portion of the participants were training in Konya, Türkiye, as part of the preparation camp for the European Senior Weightlifting Championship (April 13–21, 2025; Chisinau, Moldova), while the remaining athletes were preparing for the IWF World Junior Championship (April 30–May 5, 2025; Lima, Peru). All participants were elite level female weightlifters who had previously won medals at the European and World Championships as well as the Youth Olympic Games.

As part of the joint angle measurement protocol, assessments were conducted for cervical flexion/extension, shoulder flexion/extension, abduction, internal/external rotation, and elbow flexion. These movements were evaluated using both a digital goniometer and the Azure Kinect sensor. All measurements were performed under standard conditions between March 1 and April 5, 2025 and recorded for later analysis. Demographic characteristics, anthropometric data, and weightlifting experience of the participants are presented in Table 1.

**Table 1:** Descriptive characteristics of elite female weightlifters: Anthropometric, demographic, and performance variables.

Female weightlifting athletes (n = 21)	Parameter	Mean	Standard Deviation	Median	IQR
**Age (year)**	18,86	3,73	18.00	9.00
**Height (cm)**	157,95	6,45	158.00	9.00
**Body weight (kg)**	60,52	13,81	58.00	27.00
**BMI (kg/m**^**2**^)	24,04	4,27	23.23	8.60
**Training age (year)**	6,81	3,02	7.00	6.00
**Max snatch (kg)**	84,57	15,48	86.00	22.00
**Max clean and jerk (kg)**	103,9	19,96	107.00	30.00

BMI: Body mass index (kg/m^2^), Max Snatch: Maximum snatch weightlifting performance (kg), Max Clean and Jerk: Maximum clean and jerk weightlifting performance (kg), IQR: Inter quantile range.

All athletes included in the study were elite-level individuals who trained twice daily, six days a week, and had no serious orthopedic injuries in the past six months. Participants with a history of upper extremity or cervical spine trauma, or any structural or systemic health condition affecting joint mobility, were excluded. Inclusion and exclusion criteria were determined by a sports medicine physician through anamnesis and physical examination. Prior to the study, all participants were informed about the testing procedures, and written informed consent was obtained. Additionally, written permission was secured from those whose photographs were included for the capture and publication of images. The study was approved by the Scientific Ethics Committee of the Faculty of Medicine at Karamanoğlu Mehmetbey University (Ethical Approval No: 08-2024/16), and the experimental phase was supported by the university’s Scientific Research Projects Coordination Unit (Project Nos: 14-M-24 & 51-M-24). The research was conducted in accordance with the ethical principles outlined in the 2013 Declaration of Helsinki. Written informed consent was obtained from all participants prior to data collection. For participants under the age of 18, written informed consent was also obtained from their parents or legal guardians, as specifically required by the Scientific Ethics Committee for participation in the study. The individual in this manuscript has given written informed consent (as outlined in the PLOS consent form) to publish these case details.

All measurements were conducted in accordance with the goniometric principles described by Norkin and White [27] and the kinesiological movement standards outlined by Luttgens and Hamilton [28]. During manual assessments, joint specific test positions, the placement of fixed and movable arms, and anatomical reference points were carefully followed. To enhance reliability, each measurement was repeated three times using both a traditional digital goniometer and the Azure Kinect system; the average of the three values was used in the analysis. Athletes wore form-fitting t-shirts and their own underwear to avoid interference with body contours. Clothing was standardized to minimize its impact on measurement accuracy. All data were collected in a quiet room at room temperature, following at least six hours of rest and a five-minute light warm-up session.

**Fig 1:**
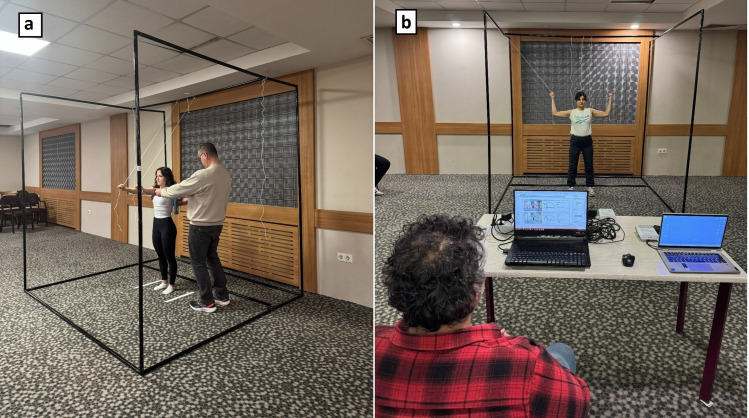
Stabilization cage used to measure joint angles. **(a)** Manual shoulder flexion measurement performed inside the stabilization cage. **(b)** Data collection during upper limb measurement with Azure Kinect in a stabilization cage.

During all measurement procedures both manual (digital goniometer) and digital (Azure Kinect) a custom designed stabilization cage was used to help participants maintain consistent positioning. This metal-pipe structure defined the physical boundaries of the testing area and provided visual alignment cues for both the participant and the examiner. Thin ropes, attached at various heights within the cage, served as support points, enabling participants to actively hold specific joint positions. This method reduced unwanted deviations and increased the standardization of posture across both manual and digital assessments (Fig 1a and 1b). The use of the stabilization system improved position repeatability, particularly for upper extremity (shoulder and elbow) and cervical measurements, while also enhancing the accuracy of Azure Kinect’s depth sensing. As a result, both manual and digital measurements were conducted under comparable postural conditions, maximizing inter-method reliability.

### Sample size calculation

The primary aim of this study was to compare two measurement methods manual goniometry and the Azure Kinect for evaluating upper extremity joint angles. Prior to participant recruitment, a pilot study involving five individuals was conducted to estimate the required sample size. In this preliminary phase, the mean shoulder extension angles were recorded as 43.4 ± 7.7 degrees with the Azure Kinect and 42.1 ± 7.8 degrees using the manual goniometer. Based on these results, the Cohen’s d effect size was calculated as 0.6, indicating a medium effect. A power analysis using this effect size revealed that a minimum of 20 participants would be required to achieve 95% confidence (α = 0.05) and 82% statistical power. The final sample consisted of 21 participants, slightly exceeding this threshold. Sample size calculations were performed using G*Power software, version 3.1.9.7.

### Azure Kinect

In this study, the Microsoft Azure Kinect sensor was employed to digitally assess joint angles of the upper extremity (shoulder and elbow) and cervical region. Azure Kinect is an advanced motion tracking system that integrates several sensing components: an RGB camera with a resolution of 3840 × 2160 pixels, a depth camera with 1024 × 1024 pixel resolution utilizing Time-of-Flight (ToF) technology, a seven-microphone audio array, and an inertial measurement unit (IMU) consisting of a three-axis accelerometer and gyroscope [29].

In this sensor, depth information is captured by a ToF depth camera, which emits modulated infrared light pulses into the environment and calculates pixel-wise distances by measuring the time delay between emission and reflection back to the sensor (*1*).


$$d=\frac{c.\Delta t}{\text{2}}$$ (1)

In Equation (1), depth values for each pixel are n computed as 3D coordinates, where c denotes the speed of light, Δt the time between light emission and reflection, and d the distance to the object.

Beyond raw depth information, Azure Kinect extracts the 3D positions of skeletal joints using a deep learning-based algorithm. During this process, depth and RGB images are spatially aligned, and the human silhouette is segmented. This enables the identification of individual body parts and the generation of joint-specific 3D coordinates in R^3^, represented as *Jointᵢ*(x*ᵢ*, y*ᵢ*, z*ᵢ*).

### Butterworth filter

The Butterworth filter is widely used in fields such as signal processing and control systems [30]. It belongs to a class of filters characterized by a maximally flat magnitude response, offering a smooth transition band and no ripples in the passband or stopband [31]. In software-based applications, its primary advantage lies in its ability to separate frequency components with minimal distortion. This property allows for the attenuation of undesired frequencies while preserving the integrity of the target frequency range. The bandwidth response of the Butterworth filter is calculated according to Equation (2) [32].


$$\left|H(jw)\right|{\mathrm{\backslash nolimits}}^{2}=\frac{\text{1}}{1+{\left(\frac{w}{w{\mathrm{\backslash nolimits}}_{c}}\right)}^{2N}}$$ (2)

In Equation (2), *|H(jω)|^2^* represents the squared magnitude of the filter’s frequency response. Here, *ω* denotes the angular frequency of the input signal, and *ωc* is the cut-off angular frequency, corresponding to the frequency beyond which the signal begins to attenuate. N indicates the order of the filter, which determines the rate of attenuation in the transition band.

The transfer function and its poles, which characterize how the frequency components of a signal are processed and determine the system’s stability, form the theoretical foundation of the Butterworth filter and explain its performance in practical applications. These are presented in Equations (3) and (4), respectively [33].


$$H(s)=\frac{\text{1}}{\prod_{K=1}^{N}\left(s-s_{k}\right)}$$ (3)

$$s_{k}=w_{c}e{\mathrm{\backslash nolimits}}^{j\frac{\pi \left(2k+N-1\right)}{2N}},k=0,1,...,N-1$$ (4)

In Equations (3) and (4), *H(s)* denotes the transfer function, s the complex frequency variable, N the number of poles, and *s_k_* the pole values corresponding to each *k*. This formulation ensures a symmetrical and balanced filter structure, resulting in the desired frequency characteristics.

Finally, as shown in Equation (5), the analog Butterworth filter was transformed into its digital form using the bilinear transform [34], preserving both stability and frequency response.


$$s=\frac{\text{2}}{T}\frac{1-z^{-1}}{1+z^{-1}}$$ (5)

In Equation (5), s denotes the complex frequency variable used in analog systems, T the sampling period, and z the frequency variable in the Z domain, commonly used in digital filter design. This transformation enables efficient processing of the signal’s frequency content, allowing for the attenuation of undesired components while preserving the integrity of the target frequency band.

### Exponentially smoothed weighted average (ESWA) filter

The ESWA filter is a nonlinear technique that applies exponentially weighted importance to past observations, smoothing time series data and minimizing the impact of short-term fluctuations. By assigning greater weight to more recent data, it responds quickly to sudden changes while effectively suppressing random variations in the signal, as shown in Equation (6) [29].


$$\theta_{t}=\alpha .X_{t}+(1-\alpha ).\theta_{t-1}$$ (6)

In Equation (6), *θt* denotes the filtered output at time t, *Xt* the new incoming data, α the smoothing factor, and *θt − 1* the previous filtered value. In the ESWA filter, the selection of the α parameter plays a critical role, as it determines the weight assigned to each new data point relative to past values. In this study, α was set to 0.6, as suggested by Del Bimbo et al. [35].

### Experimental study

The experimental procedure consisted of two stages. In the first stage, joint range of motion in the upper extremities of elite female weightlifters from the Turkish National Team was assessed using a digital goniometer (Baseline® Digital goniometer).

The evaluation included cervical flexion and extension, shoulder flexion, extension, abduction, internal and external rotation, as well as elbow flexion. Measurements for the shoulder and elbow joints were taken on both sides of the body, with three repetitions per side. Cervical region movements were measured unilaterally, also in three repetitions.

Data from the right and left sides were first recorded separately, then averaged to obtain a single value per joint for analysis. The results presented in the tables reflect these averages. In all measurements, the mean of the three repetitions was used in the final analysis.

All measurements were conducted by a physiologist with expertise in sports science assessments, while the athlete stood in a relaxed, upright position. Each measurement was carried out with attention to proper alignment within the movement plane, precise identification of anatomical reference points, and correct placement of the goniometer’s fixed and movable arms.

To ensure postural consistency, athletes were positioned inside a custom designed stabilization cage and maintained their posture by actively holding guidance ropes attached to the upper frame, which helped them preserve the targeted joint angle (Fig 1a and 1b).

The athletes’ body weights were measured in the morning, under fasting conditions, barefoot and in light clothing, using a bioimpedance analyzer (Tanita MC 580, Japan) [36]. Heights were assessed using a portable stadiometer (Seca 213, Germany), also with participants barefoot. Body mass index (BMI) was calculated as body weight divided by the square of height (kg/m^2^) [37].

The anatomical landmarks to be measured were identified in advance by an experienced practitioner, and all measurements were carried out by the same individual to ensure consistency. During assessments, athletes wore tight-fitting T-shirts and underwear to avoid interference with measurement accuracy.

The athletes’ maximum snatch and clean & jerk values, recorded during regular training, were retrieved from the official logs of the Turkish Women’s National Weightlifting Team coaches. These performance metrics were included to objectively reflect the elite level capacities of the participants and served as contextual data supporting the sample’s competitive status.

Manual measurements were performed in accordance with the goniometric protocols described by Norkin and White [27] and Luttgens and Hamilton [28]. During data collection, the fixed arm, movable arm, and central axis of the goniometer were aligned with anatomical reference points of the relevant joint, and both the test positions and participant posture were standardized in advance. All joint range of motion assessments were conducted using a digital goniometer, with three repetitions per movement, and the mean value (in degrees) was used for analysis.

#### Cervical flexion.

**Fig 2:**
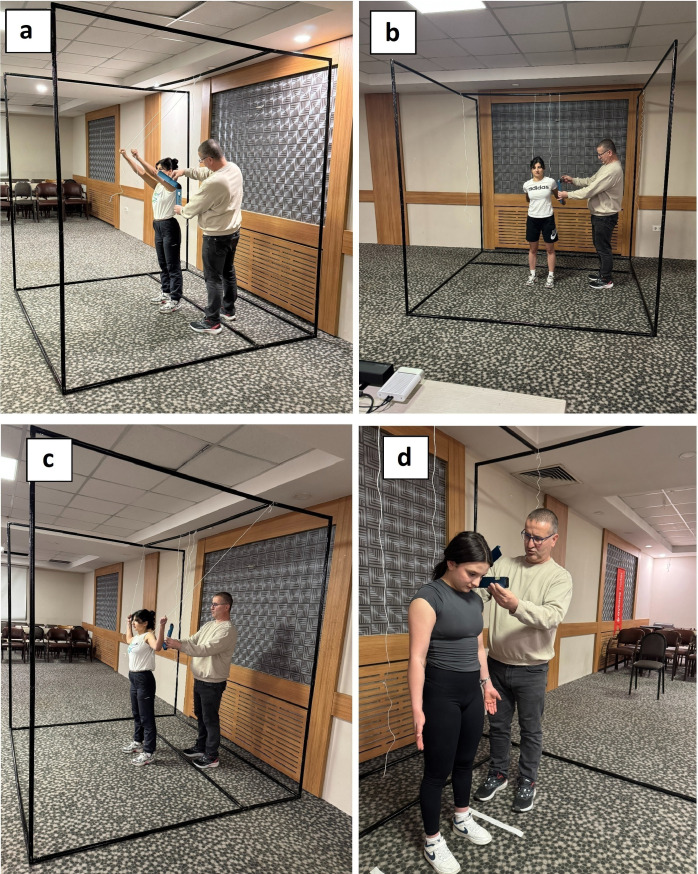
Measurement of joint angles using digital goniometry. **(a)** Shoulder flexion. **(b)** Shoulder extension. **(c)** Elbow flexion. **(d)** Cervical flexion.

While the participant stood upright, the goniometer axis was aligned with the level of the external auditory meatus, the fixed arm remained perpendicular to the floor, and the movable arm was positioned along the line of the nose. The flexion angle was recorded as the participant actively bent the head forward into full flexion (Fig 2d).

#### Cervical extension.

Participants stood in an upright position. The goniometer axis was aligned with the level of the external ear, the fixed arm remained perpendicular to the ground, and the movable arm followed the line of the nose. The extension angle was recorded as the participant tilted the head backward into full extension.

#### Shoulder flexion.

The measurement was performed with the athlete standing and raising the arm forward in the sagittal plane. The goniometer axis was positioned at the level of the humeral head, with the fixed arm aligned parallel to the trunk and perpendicular to the ground, and the movable arm positioned along the humeral shaft. The flexion angle was recorded based on the goniometer reading (Fig 2a).

#### Shoulder extension.

With the participant standing and the arm positioned alongside the trunk, the arm was moved backward in the sagittal plane. The goniometer axis was aligned with the humeral head, with the fixed arm oriented parallel to the body and perpendicular to the ground, and the movable arm aligned along the humeral shaft as the movement was performed. The extension angle was recorded in this position (Fig 2b).

#### Shoulder abduction.

With the participant standing and the test arm positioned alongside the trunk, the arm was elevated laterally within the frontal plane. The goniometer axis was aligned with the glenohumeral joint, the fixed arm oriented parallel to the torso and perpendicular to the ground, and the movable arm aligned along the humerus during the movement. The abduction angle was recorded once the arm passed shoulder level into full abduction.

#### Shoulder internal rotation.

The participant stood with the shoulder abducted to 90° and the elbow flexed to 90°, maintaining an upright posture. The goniometer axis was aligned with the olecranon process, the fixed arm remained perpendicular to the ground, and the movable arm was positioned along the forearm and tracked throughout the internal rotation movement.

#### Shoulder external rotation.

The participant stood upright with the shoulder abducted to 90° and the elbow flexed to 90°. The goniometer axis was aligned with the olecranon process, the fixed arm remained perpendicular to the ground, and the movable arm was positioned along the forearm and tracked throughout the external rotation movement.

#### Elbow flexion.

The participant stood in the anatomical position. The elbow was actively flexed from full extension (180°). The goniometer axis was aligned with the olecranon process, the fixed arm positioned along the humerus, and the movable arm aligned distally along the radius. The flexion angle was recorded across three repetitions (Fig 2c) [27,28].

In the second phase of the research, joint angles of the shoulder, elbow, and cervical regions were digitally measured using a system built with Microsoft Azure Kinect. The sensor was positioned 1.1 meters above the ground, and athletes were placed 3 meters away inside a custom-built stabilization cage. This structure, constructed from metal pipes, defined the physical boundaries of the measurement area and helped both participants and examiners maintain proper posture during data collection. Also, during this phase, the area lighting where the recordings were performed was kept within the boundaries of the indoor area without sunlight that would affect the sensor performance. Ceiling-mounted lights were chosen to minimize shadows and infrared signal interference. In measuring the illumination level, CEM DT-8820 brand-model digital luxmeter (Chinese-made device) was used. The ambient illumination level was kept at 200 lux, which is a level that will not cause deterioration in the data acquisition quality of the sensor.

**Fig 3:**
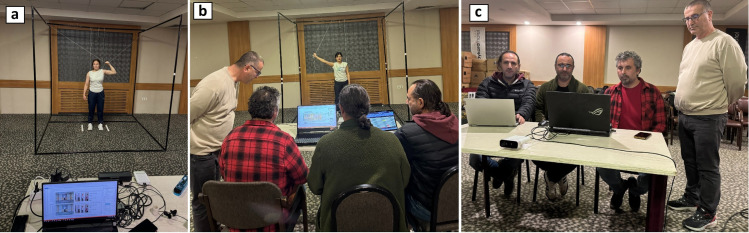
The process of measuring joint angles using Azure Kinect during the motion assessment. **(a)** An athlete positioned inside the stabilization cage, maintaining the predefined posture and prepared for the measurement process. **(b)** During shoulder abduction measurement, the athlete maintains the target posture with the support of a rope while being tracked by the Azure Kinect sensor. **(c)** Real-time monitoring of Azure Kinect sensor outputs on the computer screen during joint angle measurement.

For stabilization, thin ropes attached to the upper frame allowed participants to hold their positions during each joint movement, enhancing repeatability and measurement consistency (Fig 3).

Additionally, the layout of the system was essential for enabling the sensor to accurately detect body segments and maintain consistent participant positioning throughout the measurement.

**Fig 4:**
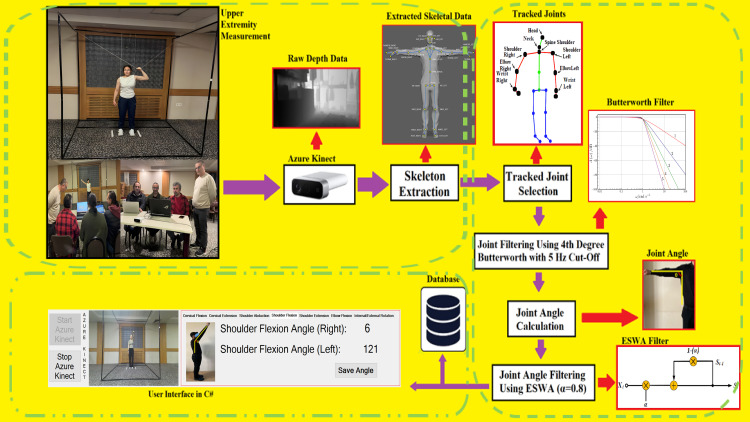
Structure and data flow of the custom-designed Azure Kinect-based joint angle measurement system.

All measurements were conducted using the standardized protocol inside the stabilization cage, under the supervision of a physiologist specialized in sports sciences. The setup and application flow are illustrated in Fig 4.

The system illustrated in Fig 4 consists of three main components. In the first part (Fig 4a), Azure Kinect was used to collect upper extremity movement data from the athlete and connected to a computer via USB-C. The data collection software was developed in C# using Visual Studio 2019 and executed on a high-performance computer (Intel Core i9 CPU, 32 GB RAM, NVIDIA RTX 4060 GPU with 8 GB VRAM). Depth and RGB image data were captured at a rate of 15 frames per second (fps). In the final step of this phase, the 3D coordinates of 32 anatomical joint points were extracted from the depth image using the Microsoft Azure Kinect Body Tracking SDK (Software Development Kit).

In the second stage of the system (Fig 4b), the first step was the selection of specific joints to be continuously tracked from those extracted in the previous phase. The 3D joint coordinate data obtained from Azure Kinect may contain pixel-level noise due to depth camera limitations. Therefore, to improve signal clarity, a fourth-order Butterworth filter with a 5 Hz cut-off frequency which corresponds to the natural frequency of human movement was applied to each selected joint separately, as also demonstrated in the study by Albert et al. [38].

Following this stage, joint angle calculations were performed using three-dimensional vector analysis and linear algebraic methods. The raw joint coordinate data collected during the measurements were first pre-processed using appropriate filters, and then used to compute joint angles. The anatomical joint pairs used for shoulder flexion/extension, abduction, internal/external rotation, and elbow flexion were defined based on validated procedures reported in prior studies involving similar digital measurement systems [15,22,39–41].

In the evaluation of cervical flexion and extension movements, the joint pairs used in this study were selected based on anatomical reference points described in the optoelectronic measurement protocol by Palmieri et al. [42]. Specifically, the 3D joint coordinates of the right and left ear, right and left shoulder, head, and neck segments which correspond to anatomical landmarks such as the bilateral anterior tragus, glabella, bilateral acromion, and the seventh cervical vertebra were used as references. These landmarks were selected due to their measurability via Azure Kinect and their consistency with validated marker placements.

Vectors between the relevant joints were computed using three-dimensional coordinate differences, and the angles between these vectors were calculated in *R^3^* space using the scalar (dot) product method [16,21,43]. For cervical region analysis in particular, angular relationships between the head, neck, and trunk segments were used as the basis for calculation. Similar methods have been applied in previous studies for cervical angle analysis using Kinect-based systems [43].

The joint angle values calculated in this study were obtained at a high temporal resolution (15 frames per second), making them challenging to interpret through visual inspection alone. To further enhance signal clarity, each joint angle series was additionally filtered using the Exponential Smoothing Weighted Average (ESWA) filter, particularly to address residual noise that may persist even after filtering the original 3D joint coordinates.

Finally, each movement was measured in three repetitions, and the average value was calculated and used for further analysis. As illustrated in Fig 4c, these averaged joint angle values were both stored in the database for subsequent statistical evaluation and displayed in real time within the user interface of the data collection software to facilitate instant observation.

All measurements obtained from the system were statistically compared with digital goniometric data, and the system’s accuracy and reliability were evaluated using correlation coefficients and Intraclass Correlation Coefficient (ICC) analysis [16,22,23]. Azure Kinect’s independence from operator skill, portability, and cost-effectiveness make it a promising alternative for joint range of motion assessments in elite-level athletes.

### Statistical analysis

Data analysis was conducted using IBM SPSS Statistics version 25.0 (IBM Corp., Armonk, NY, USA). For each numerical variable, mean, standard deviation, median, and interquartile range (IQR) were computed as descriptive statistics.

The Wilcoxon Signed-Rank Test was used to assess differences between Azure Kinect and digital goniometer measurements, under the assumption of a non-parametric distribution. The strength of association between the two measurement methods was evaluated using Spearman’s rank correlation coefficient (r).

To examine systematic bias and agreement limits between methods, Bland & Altman analysis was performed in the Python 3.7.9 environment (Python Software Foundation, DE, USA). Scatter plots with zero line for differences between Azure Kinect and Digital Goniometer in joint angle measurements of elite female weightlifters in the SPSS.

Measurement reliability and inter-method agreement were quantified using the Intraclass Correlation Coefficient (ICC). Complementary metrics such as the Coefficient of Variation (CV) and Coefficient of Repeatability (CR) were also reported.

ICC values were interpreted according to the thresholds proposed by Koo and Li [44]:

- ICC < 0.50: Poor reliability
- 0.50 ≤ ICC < 0.75: Moderate reliability
- 0.75 ≤ ICC < 0.90: Good reliability
- ICC ≥ 0.90: Excellent reliability

A significance level of p < 0.05 was adopted for all statistical tests.

## Results

The descriptive anthropometric characteristics and weightlifting performance metrics of the elite female weightlifters included in the study are summarized in Table 1. The participants had a mean age of 18.86 ± 3.73 years, with an average height of 157.95 ± 6.45 cm and body weight of 60.52 ± 13.81 kg. The average training experience was 6.81 ± 3.02 years. The best snatch and clean & jerk performances were 84.57 ± 15.48 kg and 103.90 ± 19.96 kg, respectively.

**Fig 5:**
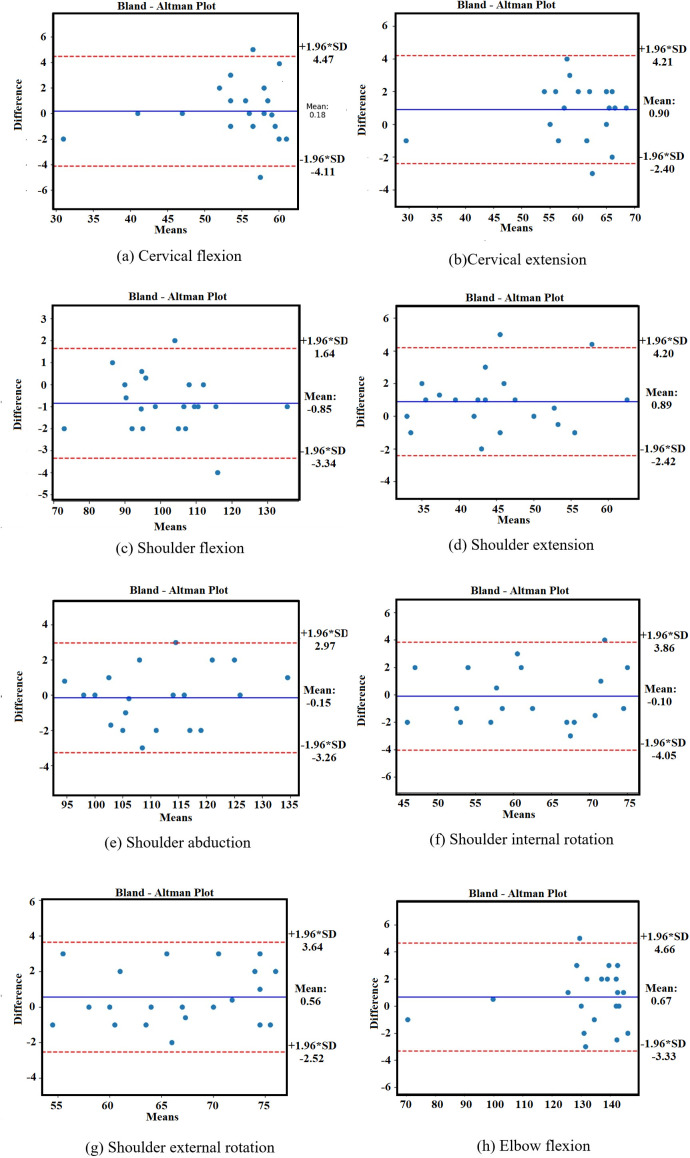
Bland–Altman plots for agreement between Azure Kinect and digital goniometer in joint angle measurements of elite female weightlifters. **(a)** Cervical flexion. **(b)** Cervical extension. **(c)** Shoulder flexion. **(d)** Shoulder extension. **(e)** Shoulder abduction. **(f)** Shoulder internal rotation. **(g)** Shoulder external rotation. **(h)** Elbow flexion.

Although the statistical comparison between Azure Kinect and the digital goniometer in measuring cervical flexion joint angles did not yield a significant difference (p = 0.774, Table 2), this alone is not sufficient to conclude equivalence. However, the strong positive correlation between the two methods (r = 0.820), the excellent agreement indicated by the intraclass correlation coefficient (ICC = 0.976, Table 3), and the narrow limits of agreement shown in the Bland–Altman analysis (mean difference = 0.18°; 95% CI: –4.11 to 4.47, Fig 5a; Table 3) together support the reliability and consistency of Azure Kinect measurements. Furthermore, the similarity in coefficients of variation (CV) and coefficients of repeatability (CR) suggests stable and comparable measurement variability between the two systems.

**Table 2:** Comparison of joint range of motion measurements between Azure Kinect and digital goniometer in elite female weightlifters.

Joint Movements	Digital Goniometer Measured Value (^0^)				Azure Kinect Measured Value (^0^)				p Value
	Mean	SD	Median	IQR	Mean	SD	Median	IQR	Median
**Cervical flexion**	54.25	6.89	56.00	6.10	54.43	7.15	56.00	6.00	.774
**Cervical extension**	59.71	7.97	62.00	9.00	60.62	8.21	61.00	9.00	**.028**
**Shoulder flexion (right/left)**	102.32	13.00	103.00	16.80	101.48	12.82	104.00	17.00	**.008**
**Shoulder extension (right/left)**	44.54	7.88	43.00	13.40	45.43	8.07	45.00	13.00	**.030**
**Shoulder abduction (right/left)**	111.24	9.74	110.00	14.20	111.1	10.09	109.00	14.00	.657
**Shoulder internal rotation (right/left)**	61.14	8.9	60.00	16.00	61.05	9.2	62.00	15.00	.859
**Shoulder external rotation (right/left)**	66.58	6.6	67.00	12.50	67.14	6.84	67.00	14.00	.144
**Elbow flexion (right/left)**	131.71	17.27	136.00	13.50	132.38	17.08	138.00	13.00	.188

SD: Standard Deviation; CV: Coefficient of Variance; CR: Coefficient of Repeatability; ICC: Intraclass Correlation Coefficient; IQR: Inter Quantile Range.

**Table 3:** Inter-method agreement and correlation of joint angle measurements in elite female weightlifters.

Measured Joint Motions	Digital Goniometer Measured Value (^0^)				Azure Kinect Measured Value (^0^)				ICC	r	Bland–Altman plot	
	Mean	SD	CV	CR	Mean	SD	CV	CR			Difference	95% CI (%)
**Cervical flexion**	54.25	6.89	12.70	19.10	54.43	7.15	13.13	19.81	.976	.820	0,18	−4,11–4,47
**Cervical extension**	59.71	7.97	13.35	22.09	60.62	8.21	13.55	22.77	.987	.917	0,90	−2,4–4,21
**Shoulder flexion (right/left)**	102.32	13.00	12.71	36.04	101.48	12.82	12.64	35.54	.997	.989	−0,85	−3,34–1,64
**Shoulder extension (right/left)**	44.54	7.88	17.69	21.84	45.43	8.07	17.76	22.36	.986	.960	0,89	−2,42–4,2
**Shoulder abduction (right/left)**	111.24	9.74	8.76	27.01	111.10	10.09	9.08	27.97	.994	.992	−0,15	−3,26–2,97
**Shoulder internal rotation (right/left)**	61.14	8.90	14.55	24.66	61.05	9.20	15.07	25.50	.988	.976	−0,10	−4,05–3,86
**Shoulder external rotation (right/left)**	66.58	6.60	9.92	18.30	67.14	6.84	10.19	18.96	.985	.960	0,56	−2,52–3,64
**Elbow flexion (right/left)**	131.71	16.85	12.79	46.70	132.38	17.08	12.90	47.35	.996	.936	0,67	−3,33–4,66

SD: Standard Deviation; CV: Coefficient of Variance; CR: Coefficient of Repeatability; ICC: Intraclass Correlation Coefficient; r: Spearman Correlation Coefficient; CI: Confidence interval (%).

Although a statistically significant difference was detected between Azure Kinect and digital goniometer measurements for cervical extension (p = 0.028, Table 2), this difference alone does not necessarily indicate a lack of agreement between the methods. A very strong positive correlation (r = 0.917) and excellent intraclass correlation coefficient (ICC = 0.987, Table 3) suggest that the two systems still provide consistent and comparable results. Bland–Altman analysis revealed a small mean difference of 0.90° with narrow 95% limits of agreement (–2.40° to 4.21°, Fig 5b), indicating minimal systematic bias. Additionally, the coefficients of variation (CV) and coefficients of repeatability (CR) were similar across both methods, supporting the stability of the measurements.

A statistically significant difference was identified between Azure Kinect and digital goniometer measurements for shoulder flexion (p = 0.008, Table 2). While this statistical difference indicates some deviation in mean values, it does not alone imply a lack of agreement between the two measurement systems. A very strong positive correlation was observed (r = 0.989), and excellent agreement was confirmed by the intraclass correlation coefficient (ICC = 0.997, Table 3). The Bland–Altman analysis showed a small mean difference of –0.85°, with 95% confidence intervals ranging from –3.34° to 1.64° (Fig 5c, Table 3). Furthermore, the similarity in coefficients of variation (CV) and repeatability coefficients (CR) across both systems indicates comparable measurement stability.

A statistically significant difference was found between digital goniometer and Azure Kinect measurements in shoulder extension (p = 0.030, Table 2). A very strong positive correlation was observed (r = 0.960), and the intraclass correlation coefficient (ICC = 0.986, Table 3) indicated excellent agreement between the methods. Bland–Altman analysis reported a mean difference of 0.89°, with 95% confidence intervals ranging from –2.42° to 4.20° (Fig 5d, Table 3). The coefficients of variation (CV) and coefficients of repeatability (CR) were similar between the two methods.

No statistically significant difference was observed between the digital goniometer and Azure Kinect in shoulder abduction measurements (p = 0.657, Table 2). A very strong positive correlation was found (r = 0.992), and the intraclass correlation coefficient (ICC = 0.994, Table 3) indicated excellent agreement between the two methods. Bland–Altman analysis showed a mean difference of –0.15°, with 95% confidence intervals from –3.26° to 2.97° (Fig 5e, Table 3). The coefficients of variation (CV) and repeatability coefficients (CR) were similar between the two methods.

No statistically significant difference was observed between the digital goniometer and Azure Kinect in shoulder internal rotation measurements (p = 0.859, Table 2). A very strong positive correlation was identified (r = 0.976), and the intraclass correlation coefficient confirmed excellent agreement between methods (ICC = 0.988, Table 3). Bland–Altman analysis revealed a mean difference of –0.10°, with 95% confidence intervals from –4.05° to 3.86° (Fig 5f, Table 3). The coefficients of variation (CV) and coefficients of repeatability (CR) were similar between the two methods.

No statistically significant difference was observed between the digital goniometer and Azure Kinect in shoulder external rotation measurements (p = 0.144, Table 2). A very strong positive correlation was identified (r = 0.960), and the intraclass correlation coefficient (ICC = 0.985, Table 3) indicated excellent agreement between the two methods. Bland–Altman analysis showed a mean difference of 0.56°, with 95% confidence intervals ranging from –2.52° to 3.64° (Fig 5g, Table 3). The coefficients of variation (CV) and coefficients of repeatability (CR) were similar between the two methods.

Although the statistical comparison between Azure Kinect and the digital goniometer in elbow flexion measurements did not show a significant difference (p = 0.188, Table 2), this alone does not establish equivalence. However, the strong positive correlation (r = 0.936), excellent intraclass correlation coefficient (ICC = 0.996, Table 3), and the small mean difference observed in Bland–Altman analysis (0.67°, 95% CI: –3.33 to 4.66, Fig 5h) together support the consistency of measurements across methods. The similar CV and CR values further reflect measurement stability.

**Fig 6:**
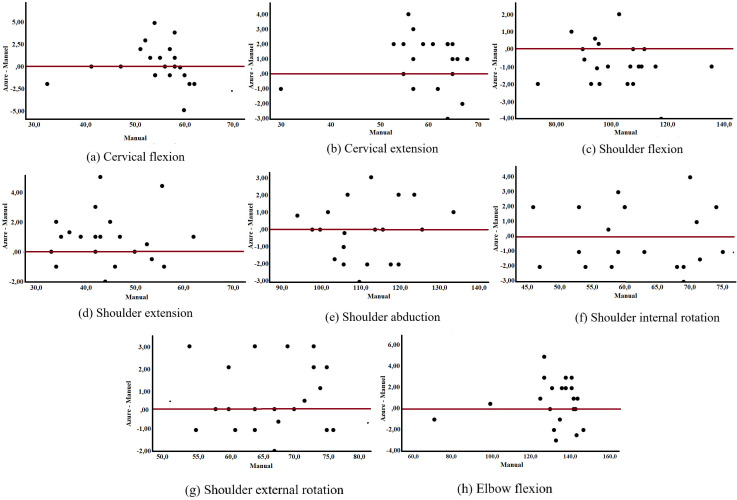
Scatter plots with fit lines comparing Azure Kinect and digital goniometer in joint angle measurements of elite female weightlifters.

Fig 6 illustrates scatter plots with fit lines comparing Azure Kinect and digital goniometer measurements across different joint movements. The plots display the distribution of paired measurements and their fitted trend lines for each movement, providing a visual representation of the correspondence between the two methods.

## Discussion

In this study, joint range of motion measurements obtained using a digital goniometer and the Azure Kinect system were compared in elite female weightlifting athletes. Eight distinct movement planes involving the cervical, shoulder, and elbow joints were analyzed. The results demonstrated strong correlations between the two methods (r = 0.820–0.992) and very high consistency (ICC = 0.976–0.997) across most joint angles. Bland–Altman analyses suggested minimal systematic differences, as indicated by low mean differences and narrow confidence intervals. Furthermore, comparable CV and CR values confirmed that both systems delivered highly stable and repeatable measurements. However, the absence of a statistically significant difference (p > 0.05) should not be interpreted as definitive evidence of equivalence between the two measurement methods. As emphasized in statistical literature, failure to reject the null hypothesis does not prove that no effect exists. Therefore, Bland–Altman plots were used as a more appropriate method to assess the agreement between devices, rather than relying solely on significance testing [45]. These overall findings suggest that Azure Kinect may be considered a valid and reliable tool for evaluating joint range of motion in the cervical and upper extremity regions, in line with the study’s primary hypothesis.

In our study, the joint range of motion data obtained using the Azure Kinect device were compared with existing findings in the literature. However, due to the limited number of scientific publications specifically addressing Azure Kinect, the discussion also incorporates validity and reliability studies involving earlier versions of Kinect, particularly Kinect V2. These systems share similar skeletal tracking algorithms and depth sensing technologies, offering a consistent methodological basis for comparison. Nevertheless, the methodological similarities between Kinect versions do not necessarily imply full measurement agreement, and findings from earlier systems should be interpreted with appropriate caution. Accordingly, the referenced sources were selected to assess potential systematic differences and to support the findings obtained in the current study.

In our study, no statistically significant difference was observed between the digital goniometer and Azure Kinect in the measurement of cervical flexion and extension movements (p = 0.774 and p = 0.028, respectively). While the correlation coefficients (r = 0.820 and 0.917) and intraclass correlation coefficients (ICC = 0.976 and 0.987) indicate a strong relationship between the two measurement methods, these metrics alone do not confirm measurement equivalence. Bland–Altman analyses, which are specifically designed to assess agreement rather than correlation, revealed low mean differences (0.18° and 0.90°) and narrow 95% confidence intervals (–4.11 to 4.47 and –2.40 to 4.21), suggesting minimal systematic bias. These findings, therefore, primarily support the reliability of Azure Kinect and its potential agreement with the goniometer, particularly as interpreted through Bland–Altman analysis.

These findings are methodologically consistent with several studies in the literature. Ryselis et al. [40] reported a 99.6% recognition accuracy for the “neck–spine mid” segment using the Kinect system, indicating high traceability and potential reliability in detecting cervical regions. Such high measurement precision may enhances the system’s applicability, particularly in spinal evaluations. Similarly, Bertram et al. [23] demonstrated a low positional error (RMSE ≈ 30 mm) and a strong correlation (r = 0.96) in tracking neck segments, suggesting promise in dynamic assessments. However, it is important to note that correlation alone does not confirm measurement agreement, and results should be interpreted considering methodological limitations. Moreover, the high-signal to-noise ratio (SNR) observed during segment recognition suggests that Kinect captures data stably and independently of environmental interferences.

These findings are broadly in line with the high correlation and low difference values observed in our study. Additional evidence of the system’s potential effectiveness in the cervical region was reported by Chatzitofis et al. [26], where a Kinect-based system was used to monitor the neck segment in real time to assess body mechanics during weightlifting. Through this method, head–neck alignment, bar trajectory, and movement precision were evaluated. While these results are promising, it should be noted that such applications rely on positional tracking rather than direct joint-level agreement analyses. Notably, this demonstrates that cervical segments can be tracked with reasonable precision not only in static postures but also during dynamic activities such as weightlifting.

Similarly, Foreman and Engsberg [46] demonstrated that the Kinect device can track the head, neck, and trunk segments with reasonably high positional precision. In their study, correlation coefficients ranging from r = 0.88 to 0.89 were reported for trunk flexion and lateral flexion movements. These findings suggest the system’s potential utility in multiplanar assessments and are methodologically aligned with the results of our study. However, it is important to interpret correlation-based findings cautiously, as correlation does not inherently indicate measurement agreement between systems.

Additionally, Sani et al. [47] reported mean position differences of 0.0831 m (X-axis) and 0.0298 m (Y-axis) when tracking the head segment using Kinect. These values indicate the potential for monitoring head–neck alignment with reasonable positional consistency, suggesting that the system may offer sufficient resolution for segment-level analysis under controlled conditions. Similarly, Sosa-León and Schwering [41] demonstrated that orientation accuracy between the head and body segments reached 95% using Azure Kinect. The same study also emphasized that skeleton data could be processed with acceptable precision, highlighting the system’s potential technical utility in sensor-based motion assessment. Nonetheless, such positional data should be interpreted carefully, as spatial accuracy does not automatically confirm measurement agreement.

Overall, the results suggest that Azure Kinect may provide acceptable reliability in both positional and orientational motion tracking, particularly under controlled conditions and when applied to gross motor segments.

When considered collectively, the findings suggest that the Azure Kinect system shows promising compatibility with traditional methods for evaluating cervical spine range of motion. While the system exhibits advantages such as segment-based tracking capability, directional resolution, and relatively low systematic deviation, these features should be interpreted within the methodological scope of the present study. Owing to its multi-dimensional measurement potential, Azure Kinect may be considered a technically useful and adaptable assessment tool, with potential applications in clinical follow-up, rehabilitation, biomechanical analysis, and sports performance monitoring pending further validation.

In our study, a statistically significant difference was observed between the digital goniometer and Azure Kinect in shoulder flexion measurements (p = 0.008). However, a very strong correlation (r = 0.989) and excellent reliability (ICC = 0.997) were found between the two methods. While this may initially appear contradictory, correlation reflects the consistency of measurement patterns rather than absolute agreement. The Bland–Altman analysis, which is more appropriate for assessing measurement agreement, showed a low mean difference of –0.85° with a narrow 95% confidence interval (–3.34° to 1.64°), suggesting minimal systematic bias. Furthermore, the similarity of CV and CR values indicates that the system provided reasonably stable and repeatable measurements under the tested conditions.

These findings are methodologically aligned with several studies in the literature. For instance, Çubukçu et al. [22] reported an ICC of 0.851 for shoulder flexion in a comparative analysis using Kinect V2 and attributed potential measurement accuracy to low deviation and narrow limits of agreement (LoA). In a subsequent telerehabilitation study conducted by the same research group, significant improvements in shoulder flexion angle were observed, and these changes were found to be strongly consistent with digital goniometer measurements [39]. These results suggest that Azure Kinect may be effective for instantaneous joint angle assessment and shows promise for tracking longitudinal changes in rehabilitation contexts. However, further validation is needed before widespread clinical adoption.

In the study by Nixon et al. [17], Kinect and Vicon systems were compared, and the average absolute error rates in shoulder flexion were found to be below 10% (right: 9.96%, left: 9.31%), indicating potential measurement consistency. Similarly, Lee et al. [16] reported ICC values of 0.864 and 0.906 for active and passive flexion movements, respectively. The mean difference was calculated as –6.86°, and the system demonstrated promising diagnostic performance, with the area under the ROC curve exceeding 0.95. Collectively, these findings suggest that Azure Kinect may offer high diagnostic sensitivity in upper extremity joint angle measurements; however, additional validation is recommended before clinical application.

A notable application of Kinect technology for evaluating wide-range, functionally significant movements was presented by Chatzitofis et al. [26] in a study on weightlifting athletes. In this study, shoulder flexion movements were recorded at a sampling rate of 30 Hz, and angular analyses yielded reasonably consistent results in both dynamic and linear motion planes. Similarly, Foreman and Engsberg [46] reported high correlations (r = 0.95–0.98) and low error margins in sagittal plane shoulder flexion measurements conducted with Kinect V2. The authors noted that ensuring trunk stability played a critical role in improving positional consistency during measurements.

However, systematic deviations in measurement accuracy have been identified in several studies. Faity et al. [48] reported an ICC of 0.50 and noted that the device overestimated shoulder flexion angles compared to the actual values. Similarly, although the mean difference was calculated as –16.6° in the study by Huber et al. [19], the ICC values remained within the 0.85–0.95 range, suggesting that the system may retain relative consistency in pattern detection despite absolute deviations. Such discrepancies may be attributed to technical and methodological factors, including device generation, participant posture variability, calibration differences, and limitations in algorithmic joint estimation sensitivity.

Oosterwijk et al. [49] reported that the average shoulder flexion required for performing daily life activities is approximately 117°, and the fact that the mean values obtained in our study closely matched this threshold suggests that Azure Kinect may have potential utility in real-world applications. Additionally, in the study by Gauthier and Cretu [50], time-series analyses of shoulder flexion and abduction revealed that the angular profiles generated by Kinect were reasonably consistent for functional evaluation. These findings support not only the system’s capacity for static measurement, but also its potential applicability in dynamic motion tracking. Finally, the vector-based calculation model proposed by Syarif et al. [51] estimated shoulder flexion angles with relatively high geometric accuracy, thereby supporting the plausibility of the underlying algorithmic approach used by Azure Kinect.

When all these findings are considered together, they suggest that even for joint movements such as shoulder flexion which involve a wide range of motion and have high functional and clinical importance the Azure Kinect system may offer reasonable measurement agreement with conventional methods. It also shows potential as a technically applicable tool for dynamic analysis and rehabilitation monitoring. Notably, studies by Çubukçu et al. [39] and Özsoy et al. [52] confirmed that Kinect-based systems can effectively track meaningful functional changes during rehabilitation. In parallel, time-series analysis results presented by Foreman and Engsberg [46] and Gauthier and Cretu [50] showed that the system may perform consistently in both instantaneous measurements and longitudinal monitoring contexts. Taken together, these aspects indicate that Azure Kinect holds promise as a multi-purpose evaluation platform for clinical and sports performance settings; however, its broader application requires further empirical validation.

In our study, a statistically significant difference was observed between the digital goniometer and Azure Kinect in measuring shoulder extension range of motion (p = 0.030). Nevertheless, the two methods demonstrated a very strong correlation (r = 0.960) and a high intraclass correlation coefficient (ICC = 0.986), which reflects consistency in rank-order measurements but does not, on its own, imply absolute agreement. The low mean difference (0.89°) and narrow 95% confidence interval (–2.42° to 4.20°) obtained from the Bland–Altman analysis indicate limited systematic bias, suggesting a reasonable level of agreement. Additionally, the similarity of the coefficient of variation (CV) and coefficient of repeatability (CR) values suggests acceptable consistency and reproducibility of the measurements under the tested conditions.

These findings are consistent with the shoulder extension results reported by Çubukçu et al. [22] using the Kinect V2 system. Although the ICC value reported in that study was moderate (0.620), the low mean difference and narrow limits of agreement (LoA) indicated a limited but acceptable level of measurement agreement. These results, while not conclusive, suggest a degree of methodological consistency that aligns with our current findings.

In a more recent investigation by the same research group, a Kinect-based telerehabilitation program was shown to significantly improve shoulder extension range, and this improvement was found to be substantially consistent with trends observed in measurements obtained from a digital goniometer [39]. Collectively, these findings indicate that the system may serve as a potentially useful tool not only for initial assessment, but also for monitoring functional changes over time in rehabilitation contexts; however, further research is recommended to confirm its clinical applicability.

In the study conducted by Oosterwijk et al. [49], the required shoulder extension range for daily living activities was reported to be between 20° and 30°. In this context, the fact that the average shoulder extension values obtained in our study were substantially higher (44°–45°) suggests that Azure Kinect is capable of capturing larger angular ranges with reasonable measurement sensitivity. This finding indicates the system’s potential for application in both clinical assessment and performance-based analysis. Taken together, these findings show that even in joint movements such as shoulder extension where segmental differentiation and trunk stability are critical to measurement accuracy Azure Kinect may provide acceptable agreement with conventional methods and offers a technically feasible measurement framework that could be considered for use in rehabilitation monitoring, functional evaluation, and sports performance analysis; however, broader clinical validation remains necessary.

In our study, no statistically significant difference was observed between the digital goniometer and Azure Kinect in shoulder abduction measurements (p = 0.657); however, this lack of significance does not conclusively indicate that the two methods are equivalent. A very strong correlation (r = 0.992) and a high intraclass correlation coefficient (ICC = 0.994) were obtained, suggesting strong relative consistency between the two systems in rank-order assessments. These findings indicate a high degree of association, though further analysis is needed to determine the extent of absolute agreement.

The minimal mean difference (–0.15°) and narrow 95% confidence interval (–3.26° to 2.97°) observed in the Bland–Altman analysis suggest limited evidence of systematic bias between the two measurement methods. Furthermore, the similarity in the coefficient of variation (CV) and coefficient of repeatability (CR) values indicates acceptable measurement consistency under the tested conditions, supporting the potential reliability of Azure Kinect in assessing shoulder abduction.

These findings are broadly consistent with the shoulder abduction measurements reported by Beshara et al. [15] using the HumanTrak system. In that study, intra-rater (ICC = 0.85) and inter-rater (ICC = 0.88) reliability values were found to be high, and the ICC value reached 0.94 during fixed-target assessments. Measurement differences were largely within the accepted clinical threshold of ±5°, suggesting the potential applicability of such systems. Similarly, Çubukçu et al. [22] reported an ICC of 0.861 for shoulder abduction measured with Kinect V2, along with a mean difference of 0.33° and limits of agreement (LoA) between –4.86° and 5.51°. These results indicate no strong evidence of systematic bias and a relatively low degree of random measurement variability.

The values obtained in our study (difference = –0.15°; 95% CI: –3.26° to 2.97°) demonstrate general consistency with previous findings, suggesting that Azure Kinect may offer acceptable measurement agreement for shoulder abduction assessment in athletic populations. While these results are promising, further validation is necessary to establish its broader applicability and measurement accuracy.

Nixon et al. [17] reported the mean absolute error rates between the Kinect and Vicon systems as 8.18% (right) and 10.0% (left) for shoulder abduction, which fall within the range considered acceptable in previous motion analysis studies. Despite being a markerless motion capture system, Kinect demonstrated sufficient precision to suggest potential applicability in home-based rehabilitation and functional movement contexts. Similarly, Lee et al. [16] reported ICC values of 0.911 for active and 0.876 for passive shoulder abduction measured with Kinect, with a mean difference of –5.10°. These values are within literature-based thresholds and suggest a reasonable level of consistency between Kinect and goniometric measurements. Furthermore, ROC analysis results indicated that Kinect may provide useful diagnostic discrimination for shoulder abduction assessment.

In a study by Çubukçu et al. [39], a 30.42% improvement in shoulder abduction range was observed following a telerehabilitation program utilizing Kinect, and this improvement was found to correspond closely with digital goniometer measurements, suggesting a high degree of measurement consistency. These findings suggest that Kinect may serve as a potentially useful platform progress monitoring during treatment. In contrast, Faity et al. [48] reported a low ICC value (0.29) for shoulder abduction measured with Kinect V2, noting a reduction in accuracy particularly in overhead positions. However, the high ICC value (0.994), minimal difference, and narrow confidence interval obtained in the current study indicate that Azure Kinect may address some of these technical limitations, potentially offering acceptable precision even in challenging joint positions.

In the study by Foreman and Engsberg [46], a correlation coefficient of r = 0.91–0.96 was reported between the Kinect V2 device and the reference system for shoulder abduction, and the authors noted that measurement accuracy improved particularly in abduction movements performed in the lateral plane. These findings demonstrate methodological similarity with the high correlation and limited deviation values reported in our study, suggesting a comparable trend in shoulder abduction measurements. Similarly, Huber et al. [19] reported that the Kinect system yielded an ICC of 0.76 for shoulder abduction, with a mean difference of –1.5°. The limits of agreement (LoA) ranging from –7.0° to 4.1° fall within previously reported thresholds, indicating that the system may yield reasonable measurement differences, particularly in frontal plane abduction tasks.

Finally, Oosterwijk et al. [49] reported that the average shoulder abduction range of motion required for daily living activities in healthy individuals is 82°–90°, with maximum values ranging from 110° to 120°. In the present study, the average shoulder abduction value was approximately 111°, which suggests that Azure Kinect may be capable of capturing functionally relevant joint angles not only in controlled laboratory conditions, but also in alignment with real-world functional demands. Taken together, the high correlation coefficient, excellent ICC value, minimal measurement difference, and narrow confidence interval obtained in this study suggest that Azure Kinect may offer a statistically consistent and technically viable method for assessing shoulder abduction range of motion in specific populations.

In this context, the findings suggest that Azure Kinect shows a reasonable level of consistency with traditional measurement methods for shoulder abduction and may offer adequate sensitivity and repeatability for both clinical and functional applications. When its technical characteristics are considered alongside its observed measurement performance, Azure Kinect may serve as a complementary method for upper extremity assessments. Moreover, when interpreted in conjunction with prior research on rehabilitation and clinical monitoring [39,52], these results indicate the system’s potential applicability in both healthcare-related monitoring and sports performance evaluation.

No statistically significant difference was found between the digital goniometer and Azure Kinect in shoulder internal rotation measurements (p = 0.859). The strong correlation coefficient (r = 0.976) and excellent ICC value (0.988) suggest a high degree of measurement consistency between the two methods. Furthermore, the minimal deviation (difference = –0.10) and narrow confidence interval (95% CI: –4.05 to 3.86) obtained from the Bland–Altman analysis indicate minimal potential for systematic bias. The similarity observed in the coefficient of variation (CV) and coefficient of repeatability (CR) values suggests that the system yields stable and repeatable measurements for shoulder internal rotation.

These findings are consistent with the shoulder internal rotation results reported by Çubukçu et al. [22], where the ICC value was 0.965. The authors suggested that the system may provide clinically acceptable consistency, attributable to low measurement differences and narrow limits of agreement. Similarly, a more recent study by the same research group reported a 19.63% improvement in shoulder internal rotation during a Kinect-based rehabilitation protocol, with a close correspondence observed between Kinect and digital goniometer measurements [39].

In the study conducted by Özsoy et al. [52], shoulder internal rotation measurements demonstrated high reliability, with an ICC value of 0.75. Additionally, the standard error of measurement (SEM = 4.39) and minimal detectable change (MDC = 12.16) were found to be within clinically acceptable ranges, suggesting that the system may be sensitive to small changes in joint angles. Although the narrow range of motion inherent in internal rotation typically requires greater measurement sensitivity, the authors reported that Kinect was able to detect such changes. These findings are consistent with the high correlation and low systematic deviation observed in the present study, indicating potential applicability of Azure Kinect for joint angle monitoring in internal rotation tasks.

Ryselis et al. [40] reported that the Kinect system was observed to show improved angular resolution and segment tracking performance, particularly in multi-camera or multi-environment configurations.

The authors emphasized that complex joint actions occurring in close proximity to the torso, such as shoulder internal rotation, present a critical test for the system’s segment detection capability. Despite this challenge, the study reported that the skeletal modeling algorithms and angular measurement precision offered by Kinect may be sufficient for certain clinical applications.

Taken together, these findings suggest that Azure Kinect demonstrates statistical consistency in shoulder internal rotation measurements and may provide adequate angular resolution and measurement stability for assessing complex axial joint movements. This consistency particularly in near-body plane motions such as internal rotation, which are challenging to capture indicates the system’s potential utility for precise segment tracking. Therefore, Azure Kinect may serve as a modern and adaptable platform with promising applications in clinical monitoring, athlete performance evaluation, and advanced rehabilitation contexts.

In this study, no statistically significant difference was observed between the digital goniometer and Azure Kinect in shoulder external rotation range of motion measurements (p = 0.144). The high correlation coefficient (r = 0.960) and Intraclass Correlation Coefficient (ICC = 0.985) suggest a high degree of relative consistency between the two methods. Additionally, the minimal deviation (difference = 0.56) and narrow confidence interval (95% CI: –2.52 to 3.64) observed in the Bland–Altman analysis indicate that minimal systematic bias was present. The similarity in the coefficient of variation (CV) and coefficient of repeatability (CR) values across both systems further suggests stable and repeatable measurements.

These findings are consistent with the shoulder external rotation results reported in the Kinect V2-based study by Çubukçu et al. [22], which recorded an ICC value of 0.874 for this movement. The study also noted that the mean difference was low and the limits of agreement (LoA) were narrow and within clinically acceptable ranges. The research demonstrated that both digital and markerless systems produced comparable measurement trends, even in complex axial movements such as shoulder external rotation. In line with these findings, the present study suggests that Azure Kinect may be a technically and statistically appropriate too for assessing such motions.

In the comparative study conducted by Lee et al. [16], the Intraclass Correlation Coefficient (ICC) between Kinect and goniometric measurements was reported as 0.892 for active and 0.841 for passive external rotation movements. The mean difference was –5.94°, which was considered clinically acceptable. Furthermore, the Receiver Operating Characteristic (ROC) analysis uggested the system’s potential for diagnostic utility in capturing rotational joint angles. These findings suggest that Azure Kinect may be a potentially appropriate tool for assessing joint movements requiring high angular precision, such as shoulder external rotation.

In a telerehabilitation-based study by Çubukçu et al. [39], the Kinect system demonstrated a high level of agreement in shoulder external rotation measurements, and the post-treatment angular improvements were reported to be greater than those observed in conventional treatment groups. These findings highlight the potential of the system not only for assessment but also for longitudinal monitoring applications. Similarly, in the study by Huber et al. [19], a very high ICC value (0.98) was obtained between Kinect and a marker-based reference system for external rotation. However, the limits of agreement (LoA) were relatively wide (–14.3° to 16.9°), suggesting greater inter-individual measurement variability. The authors attributed this to the 3D anatomical complexity of the shoulder and limitations in depth resolution. In contrast, the narrower difference range and LoA values found in the present study suggest that Azure Kinect may offer relatively consistent measurements for this specific rotational movement.

Özsoy et al. [52] reported an ICC value of 0.67 for shoulder external rotation, with a Standard Error of Measurement (SEM) of 5.16° and a Minimal Detectable Change (MDC) of 14.31°. Although this suggests relatively high measurement variability, the study concluded that the overall reliability remained within clinically acceptable boundaries. In contrast, the lower mean difference and narrower limits of agreement (LoA) observed in the current study may indicate that Azure Kinect offers relatively lower measurement variability for this specific rotational axis. Similarly, Ryselis et al. [40] reported high reliability (ICC > 0.89) even in complex angular motions in evaluations involving multi-sensor Kinect installations. That study emphasized the system’s success in skeletal tracking and test–retest reliability, particularly for movements requiring three-dimensional axis rotations, such as external rotation. The findings from the present study are consistent with these prior observations and suggest the potential utility of Azure Kinect in both static and dynamic external rotation assessments.

When considered collectively, these suggest that Azure Kinect may deliver precise and stable joint angle measurements even in multi-planar movements influenced by segmental complexity, such as shoulder external rotation. This performance suggests that the system may be suitable not only suitable for standard clinical assessments, but also has potential utility in dynamic performance monitoring and advanced rehabilitation scenarios, where high angular resolution and movement consistency are required.

In this study, no statistically significant difference was found between digital goniometer and Azure Kinect measurements for elbow flexion range of motion (p = 0.188). The results revealed a strong correlation coefficient (r = 0.936) and an excellent reliability index (ICC = 0.996), suggesting a high degree of relative consistency between the two methods. Furthermore, the low mean difference (0.67°) and narrow confidence interval (95% CI: –3.33° to 4.66°) observed in the Bland–Altman analysis indicate the absence of substantial systematic bias. The similarity in coefficient of variation (CV) and coefficient of repeatability (CR) values further further suggests that both systems produce stable and repeatable measurements.

These findings are methodologically aligned with results reported in prior studies. Chen and Wei [21] demonstrated that Azure Kinect is capable of delivering high-resolution angular data for the elbow joint by utilizing vector-based analysis derived from 3D skeletal tracking algorithms. Similarly, Bertram et al. [23] reported high correlation coefficients (r = 0.95–0.98) and strong agreement levels (ICC = 0.94) when comparing Azure Kinect with the marker-based Qualisys system, which is consistent with its use in elbow joint motion analysis. In another relevant study, Chatzitofis et al. [26] evaluated elite weightlifting athletes and noted that Kinect successfully tracked elbow and shoulder joint movements at 30 Hz, providing potentially reliable data for performance assessment. Additionally, Foreman and Engsberg [46] reported that the Kinect system could accurately track elbow flexion and extension during functional reaching tasks, demonstrating a high correlation with video-based motion capture systems (r = 0.94–0.99).

While Faity et al. [48] noted systematic deviations in Kinect v2 measurements, the low difference values observed in the present study suggest that such deviations may be reduced with Azure Kinect, particularly in elbow flexion measurements. Similarly, Özsoy et al. [52] reported high reliability (ICC = 0.77) and low measurement deviation in their comparative assessment of elbow flexion, which aligns with the present study’s findings regarding measurement stability.

These findings are consistent with the outcomes of the present study. Gauthier and Cretu [50] demonstrated that the Kinect sensor can reliably track complex joint segments, such as elbow movements, on a time-series basis, enabling accurate angular motion analysis. From a technical standpoint, the vector-based computation system developed by Syarif et al. [51] further supports the algorithmic validity of Azure Kinect by accurately estimating elbow range of motion through the geometric relationships between the shoulder, elbow, and wrist segments. The high correlation coefficient and ICC value obtained for elbow flexion, combined with the low mean difference and narrow confidence interval, suggest that Azure Kinect may offer not only statistical consistency but also potential technical precision and functional reliability. These characteristics indicate its potential utility as a promising tool for joint motion assessment in both clinical practice and sports performance applications. While the present study focused on a homogeneous sample of elite female weightlifting athletes, the technical features of the Azure Kinect system such as markerless skeletal tracking and multi-segment analysis suggest that its application may extend to broader populations. These include individuals from different age groups, sexes, and sporting or rehabilitative contexts. However, the generalizability of these findings requires further investigation through studies involving more diverse participant groups and functional tasks.

## Limitations

This study was limited to elite female athletes from the Turkish National Women’s Weightlifting Team, which restricts the generalizability of the findings to other populations such as different age groups, genders, or sports disciplines. The evaluation focused on a limited number of joint range of motion tasks, excluding complex or multi-planar movements, which restricts broader interpretations of Azure Kinect’s biomechanical analysis capabilities. All measurements were conducted under controlled laboratory conditions with stabilized postural alignment, and therefore may not fully reflect the system’s performance in real-life scenarios or during dynamic tasks. Moreover, since weightlifting is a high-load, dynamic sport, future studies should explore the validity and reliability of Azure Kinect under loaded, functional movement conditions. Additionally, as skeleton tracking algorithms and hardware versions of Azure Kinect continue to evolve, the findings are specific to the current generation of the system. Finally, all assessments were performed by a single evaluator, and thus inter-rater reliability was not assessed. As a result, conclusions regarding cross-user consistency remain limited.

## Conclusions

The findings of this study revealed that the Azure Kinect system demonstrated strong agreement with the digital goniometer in measuring joint range of motion across both the cervical region and upper extremity. The low measurement deviation and narrow confidence intervals observed in the Bland–Altman analyses indicated the absence of systematic bias, while the similarity in the coefficients of variation and repeatability confirmed the stability and consistency of the measurements. Based on these results, Azure Kinect can be considered a statistically valid and reliable tool for assessing joint mobility in elite female weightlifters, offering potential use in both clinical and athletic performance settings.

## Practical applications

The findings of this study demonstrate that the Azure Kinect system provides high correlation and excellent reliability in the assessment of cervical and upper extremity joint range of motion. With its low deviation rates, narrow confidence intervals, and high repeatability, the system serves as a technically robust tool for both real-time assessment and longitudinal monitoring in medical and sports applications. Owing to its portability and cost-effectiveness, Azure Kinect offers an accessible and standardized solution, particularly valuable in time-constrained or resource-limited settings, enabling fast and accurate movement analysis in elite-level athletes. Furthermore, comparing the measured joint angle data with the ideal range of motion required for fundamental lifting techniques such as the snatch and clean and jerk may provide coaches with valuable reference points for enhancing technical performance and reducing injury risk.

## Supporting information

S1 File S2_Code (txt)
